# SYNJ2 is a novel and potential biomarker for the prediction and treatment of cancers: from lung squamous cell carcinoma to pan-cancer

**DOI:** 10.1186/s12920-022-01266-0

**Published:** 2022-05-17

**Authors:** Wei Hou, Guo-Sheng Li, Li Gao, Hui-Ping Lu, Hua-Fu Zhou, Jin-Liang Kong, Gang Chen, Shuang Xia, Hong-Yu Wei

**Affiliations:** 1 Guangxi Key Laboratory of Thalassemia Research, Life Sciences Institute, Guangxi Medical University, Nanning, 530021 Guangxi Zhuang Autonomous Region People’s Republic of China; 2 Key Laboratory of Thalassemia Medicine, Chinese Academy of Medical Sciences, Nanning, 530021 Guangxi Zhuang Autonomous Region People’s Republic of China; 3 Department of Pathology, The First Affiliated Hospital of Guangxi Medical University, Nanning, 530021 Guangxi Zhuang Autonomous Region People’s Republic of China; 4 Department of Cardiothoracic Surgery, The First Affiliated Hospital of Guangxi Medical University, Nanning, 530021 Guangxi Zhuang Autonomous Region People’s Republic of China; 5 Ward of Pulmonary and Critical Care Medicine, Department of Respiratory Medicine, The First Affiliated Hospital of Guangxi Medical University, Nanning, 530021 Guangxi Zhuang Autonomous Region People’s Republic of China; 6 Department of Human Anatomy, Guangxi Medical University, Nanning, 530021 Guangxi Zhuang Autonomous Region People’s Republic of China; 7 Department of Organic Chemistry and Medicinal Chemistry, Pharmaceutical College, Guangxi Medical University, Nanning, 530021 Guangxi Zhuang Autonomous Region People’s Republic of China

**Keywords:** Gene expression, Prognosis, Prediction, Treatment, Biomarker, Immune

## Abstract

**Background:**

The roles and clinical values of synaptojanin 2 (*SYNJ2*) in lung squamous cell carcinoma (LUSC) remain unclear.

**Methods:**

A total of 2824 samples from multi-center were collected to identify the expression of *SYNJ2* in LUSC by using Wilcoxon rank-sum test, *t*-test, and standardized mean difference (SMD), and 194 in-house samples were also included to validate SYNJ2 expression in LUSC. The clinical roles of *SYNJ2* were investigated via receiver operating characteristic (ROC) curves, univariate Cox regression analysis, and Kaplan–Meier plots. The underlying mechanisms of *SYNJ2* in LUSC were explored by gene set enrichment analysis and immune correlation analysis. Further, a pan-cancer analysis based on 10,238 sapiens was performed to promote the understating of the expression and clinical significance of *SYNJ2* in multiple human cancers.

**Results:**

SYNJ2 was found to be significantly upregulated in LUSC at both mRNA and protein levels (*p* < 0.05, SMD = 0.89 [95% CI 0.34–1.45]) via public and in-house samples. Overexpressed *SYNJ2* predicted poor prognosis for LUSC patients (hazard ratio = 2.38 [95% CI 1.42–3.98]). The cancer-promoting effect of *SYNJ2* may be related to protein digestion and absorption and extracellular matrix-receptor interaction. *SYNJ2* expression was closely related to immune cell infiltration, indicating its role in the immune response. Moreover, the distinct expression levels and essential clinical relevance of *SYNJ2* in a series of cancers were initially revealed in this study.

**Conclusions:**

This study disclosed the clinical significance of SYNJ2 in LUSC and multiple cancers, demonstrating the novel and potential biomarker for predicting and treating cancers.

**Supplementary Information:**

The online version contains supplementary material available at 10.1186/s12920-022-01266-0.

## Introduction

Lung cancer is the most lethal malignant tumor in the world. As many as 1,796,144 people died of lung cancer worldwide in 2020, accounting for 18% of cancer-derived deaths [1, 2]. Lung squamous cell carcinoma (LUSC) is a common subtype of lung cancer characterized by a deficiency of known driver genes, late diagnosis, and high heterogeneity. Almost 50% of LUSC patients have developed metastases at diagnosis. The 5-year survival rates of LUSC patients with stages II, III, and IV disease are 32%, 13%, and 2%, respectively [3]. Molecular targeted therapy and immunotherapy are the main current treatment options to reduce LUSC mortality [4]. Progress in understanding the driver genes and drug resistance after systemic therapy is very slow.

A deficient endocytosis pathway is highly correlated with the development of tumor cells and drug tolerance [5]. Synaptojanin 2 (*SYNJ2*), a member of the polyphosphate 5-phosphatase family, inhibits clathrin-mediated endocytosis and functions in distinct ways compared to SYNJ1, despite the high degree of homology in their catalytic domains [6]. Genetic variation in *SYNJ2* is widely found in human diseases, such as reduced cognitive ability [7], Alzheimer’s disease and depression [8], medulloblastoma [9], colorectal cancer [10], prostate cancer [11], hairy cell leukemia [12], glioma [13], and breast cancer [14]. Hereinto, *SYNJ2* plays a critical role in glioma and breast cancer metastasis. In terms of lung cancer, only one study showed the relationship between *SYNJ2* and lung cancer; after studying 1404 lung cancer patients, it revealed that people with high *SYNJ2* transcript levels are prone to shorter survival times. The limitation of this study is that the authors only used the Kaplan–Meier Plotter [15], ignoring the heterogeneity among various subtypes of lung cancer, and based results on partial transcriptomic data [14]. Moreover, to our knowledge, the roles of *SYNJ2* in LUSC have not been reported before. Therefore, the roles and biofunctions of *SYNJ2* in LUSC remain largely unknown.

In this study, the mRNA and protein expression levels of SYNJ2 in LUSC were systematically investigated. Integrated analyses of 2824 samples from multiple databases, such as The Cancer Genome Atlas (TCGA), Gene Expression Omnibus, and ArrayExpress, were performed to understand the expression pattern of *SYNJ2* in LUSC. *SYNJ2* mRNA expression was assessed with in-house microarray, and its protein level in in-house LUSC tissue microarray samples was assessed using immunohistochemistry (IHC). Predictive and prognostic values were evaluated via receiver operator characteristic (ROC) curves and hazard ratio (HR), and the latent molecular mechanism was explored by functional enrichment with gene ontology and Kyoto Encyclopedia of Genes and Genomes (KEGG) [16–18]. Finally, a pan-cancer analysis based on 9781 samples was also performed in this study, contributing to the understanding of *SYNJ2* in multiple cancers.

## Materials and methods

This study was authorized by the Ethics Committee of The First Affiliated Hospital of Guangxi Medical University (No. 2015-KY-NSFC-019). In-house samples were obtained with the informed consent of the corresponding patients.

### Datasets collection

For LUSC-related datasets, the data collection workflow is shown in Additional file 1. Using search keyword terms “lung AND (squamous OR NSCLC OR [non-small cell]) AND (mRNA OR gene),” publicly available high-throughput RNA sequencing and microarray data were obtained from the following databases: TCGA, Gene Expression Omnibus, ArrayExpress, Sequence Read Archive, Oncomine, PubMed, and Google Scholar. The inclusion criteria for datasets were as follows: (1) *Homo sapiens*-related study; (2) Microarrays or RNA-Seq datasets; and (3) Clear subtype of lung cancer. The exclusion criteria were as follows: (1) Repeated samples between datasets; (2) Unclear subtype of lung cancer samples; and (3) Unavailable raw data. As a result, 37 datasets (Additional file 2) containing 1435 LUSC and 1428 non-LUSC samples were included in this study. Moreover, six datasets involving 323 LUSC cases were obtained to explore whether the *SYNJ2* expression was related to the overall survival (OS) of LUSC patients.

CCLE [19] database collects data from numerous cell lines of homo sapiens. The data (containing 457 samples) of the CCLE database was utilized to investigate the difference in *SYNJ2* expression between 14 kinds of cancer lines. Datasets of 32 cancers from TCGA were downloaded from the Xena database on November 16, 2021, and 9054 cancer samples and corresponding 727 control samples (Additional file 3) were included for further analysis.

### In-house mRNA microarray collection and experiment

As previously described [20], the total RNA of six in-house LUSC and control samples was extracted with TRIzol® Regent (Invitrogen, USA). The RNA purity and integrity were assessed using the OD260/OD280 ratio and the standard range of 1.8–2.1. The OD260/OD280 was greater than 1.8, and RNA was not degraded. Then the cDNA samples were synthesized and labeled. A Nanodrop ND-1000 (Agilent, California, USA) was used to detect the efficiency of fluorescent labeling. Then the chip (Aksomics Inc., Shanghai, China) was hybridized with the labeled probe under standard conditions. Finally, the results were converted into digital data for storage and analysis after scanning with an Agilent Microarray Scanner (Agilent p/n G2565BA).

### In-house tissue microarrays collection and IHC experiment

The four tissue microarray sections (No. LUC1021, LUC1501, LUC1601, and LUC2281) used in this study were produced by Fanpu Biotech, Inc. (Guilin, China). They contained 167 LUSC and 21 non-cancerous tissues, collected from August 2017 to October 2017. All accepted samples had never been exposed to chemical therapy and radiotherapy and had been diagnosed as LUSC independently by two pathologists according to the World Health Organization (2015) lung tumor histological criteria.

The SYNJ2 protein was detected using the EnVision system (Mxim, Fuzhou, China), a sensitive two-step immunohistochemical technique. All procedures were performed according to the manufacturer’s instructions. Briefly, the antigen was prepared with ethylene diamine tetraacetic acid (pH 9.0), the primary antibody (anti-SYNJ2 rabbit anti-human polyclonal antibody, orb513930, Biorbyt, UK, 1:100 dilution) was incubated at 4℃ overnight. Horseradish peroxidase-labeled secondary antibody (ready-to-use, Long Island Antibody, Shanghai, China) was added and left to react at room temperature for 25 min. Protein was visualized using 3–3′-Diaminobenzidine (DAB, Maxin, Fuzhou, China). Two pathologists independently evaluated the slides following the scoring criteria described previously [21]. In detail, a score for staining degree equals 0, 1, 2, and 3 for no, weak, moderate, and strong staining, respectively; a score for the percentage of positive cells equals 0, 1, 2, 3, and 4 for < 26%, 26–50%, 51–75%, and > 75% positive staining cells, respectively. The ultimate SYNJ2 protein level was calculated by the product of the score for staining degree and the score for the percentage of positive cells.

### Data preparation

Datasets collected from the public databases were normalized with quantile and log_2_(x + 1) using the “oligo” and “limma” packages [22, 23]. By removing batch effects using the “SVA” package [24], the 37 datasets included in this study were classified into 13 merged datasets based on the same platform (Additional file 2). Notably, the dataset “GSE6044” did not include *SYNJ2* expression and was used only to explore the differential expression genes (DEGs) between LUSC and non-LUSC groups.

### The SYNJ2 expression and its clinical relevance in LUSC

Wilcoxon rank-sum test, *t*-test, and standardized mean difference (SMD) were used to compare the difference in *SYNJ2* expression between LUSC and non-LUSC groups. When the data of a merged dataset were normally distributed and with homogeneity of variance, the *t*-test should be applied; otherwise, the Wilcoxon rank-sum test would be utilized. SMD calculation was used for the integrative analysis of *SYNJ2* expression between LUSC and non-LUSC groups. A random-effects model was established to calculate SMD, as the *I*^2^ value of the *I*^2^ test > 50%. The publication bias and robustness of SMD were evaluated by *Begg’s* test [25] and sensitivity analysis, respectively.

The area under the curve (AUC) of (ROC) curves and summary ROC curves was calculated for detecting the accuracy of *SYNJ2* mRNA expression in distinguishing LUSC samples from non-LUSC samples. The univariate Cox regression analysis was used to explore the relevance of *SYNJ2* expression to the prognosis of LUSC patients.

### The biofunctions and mechanisms of SYNJ2 in LUSC

DEGs were defined with the absolute value of log_2_ (fold change) ≥ 1. The upregulated DEGs (Up-DEGs) of LUSC were DEGs with SMD > 0, while the downregulated DEGs (Down-DEGs) were DEGs with SMD < 0. All genes from all datasets above were analyzed for correlation with *SYNJ2*. Genes meeting the criteria of *Spearman* correlation coefficient ≥ 0.3 and *p* < 0.05 were identified as positively co-expressed genes of *SYNJ2* (*SYNJ2*-PCEGs) in at least two raw datasets, while *Spearman* correlation coefficient ≤ –0.3 and *p* < 0.05 were identified as negatively co-expressed genes of *SYNJ2* (*SYNJ2*-NCEGs). The upregulated positively co-expressed genes (Up-PCEGs) were obtained by the intersection of Up-DEGs and *SYNJ2*-PCEGs, and the downregulated negatively co-expressed genes (Down-NCEGs) were determined via the intersection of Down-DEGs and *SYNJ2*-NCEGs. The Up-PCEGs and Down-NCEGs were analyzed and visualized using the “clusterprofiler” package [26] that included items from the gene ontology analysis and KEGG [27] signal pathways (the enrichment items with adjusted *p*-value < 0.05 were regarded as significant items). CIBERSORT [28] was used to detect the correlation between *SYNJ2* expression and infiltration levels of 22 types of immune cells.

### The pan-cancer analysis of SYNJ2

The Wilcoxon rank-sum tests were utilized to evaluate the differential expression of *SYNJ2* in multiple cancers. The AUC of ROC and summary ROC curves was also used to calculate the accuracy of *SYNJ2* mRNA expression in distinguishing cancer samples from their control samples. Both the univariate Cox regression analysis and Kaplan–Meier curves were used to explore the relevance between *SYNJ2* expression and the prognosis of cancer patients.

Using gene set enrichment analysis, KEGG signaling pathways of 32 cancers were explored, and the grouping criteria was the median levels of *SYNJ2* expression. TIMER database [29] provided infiltration levels of several types of immune cells for patients included in TCGA, and the data were obtained for investigating the immune relevance of *SYNJ2* in pan-cancer. Both tumor mutational burden (TMB) and microsatellite instability (MSI) data were downloaded from the research of Liu et al. [30], and the expression levels of 46 immune checkpoints were from the TCGA dataset included in this study.

### Statistical analysis

**Fig. 1:**
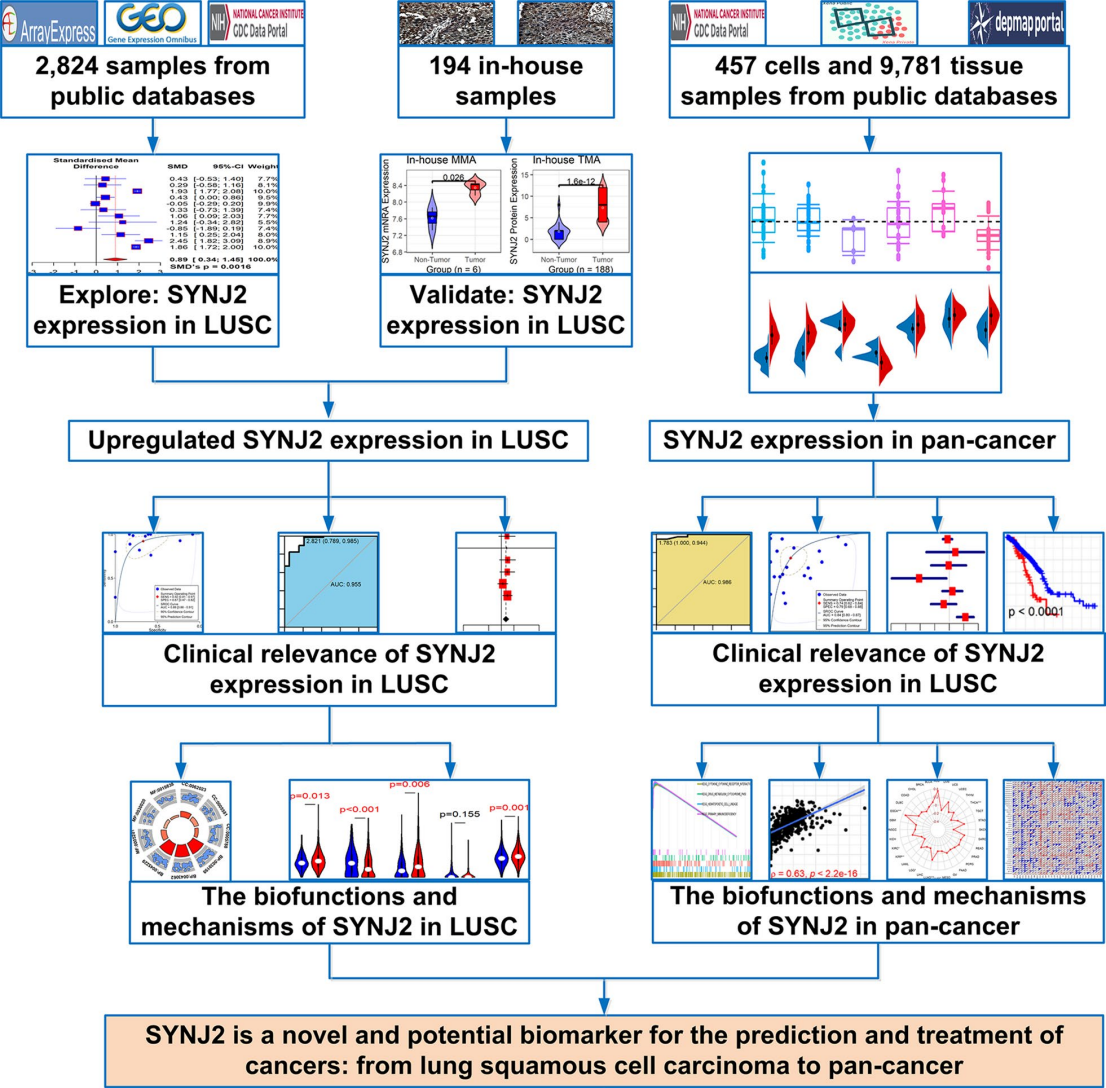
The design of this study. LUSC, lung squamous cell carcinoma

In addition to the statistical methods mentioned above, the *Spearman* correlation coefficient was utilized in all the correlation analyses for detecting the immune correlation of *SYNJ2* in pan-cancer. An SMD value was considered significant if the corresponding 95% confidence interval (CI) did not exclude zero, while *p* < 0.1 indicated significant publication bias of SMD results. For HR, that 95% CI did not include 1 or *p* < 0.05 suggested statistical significance. All calculating processes and figures of this study were completed in R software (v4.1.0). The design of this study can be viewed in Fig. 1.

## Results

### Upregulated SYNJ2 expression was identified in LUSC

**Fig. 2:**
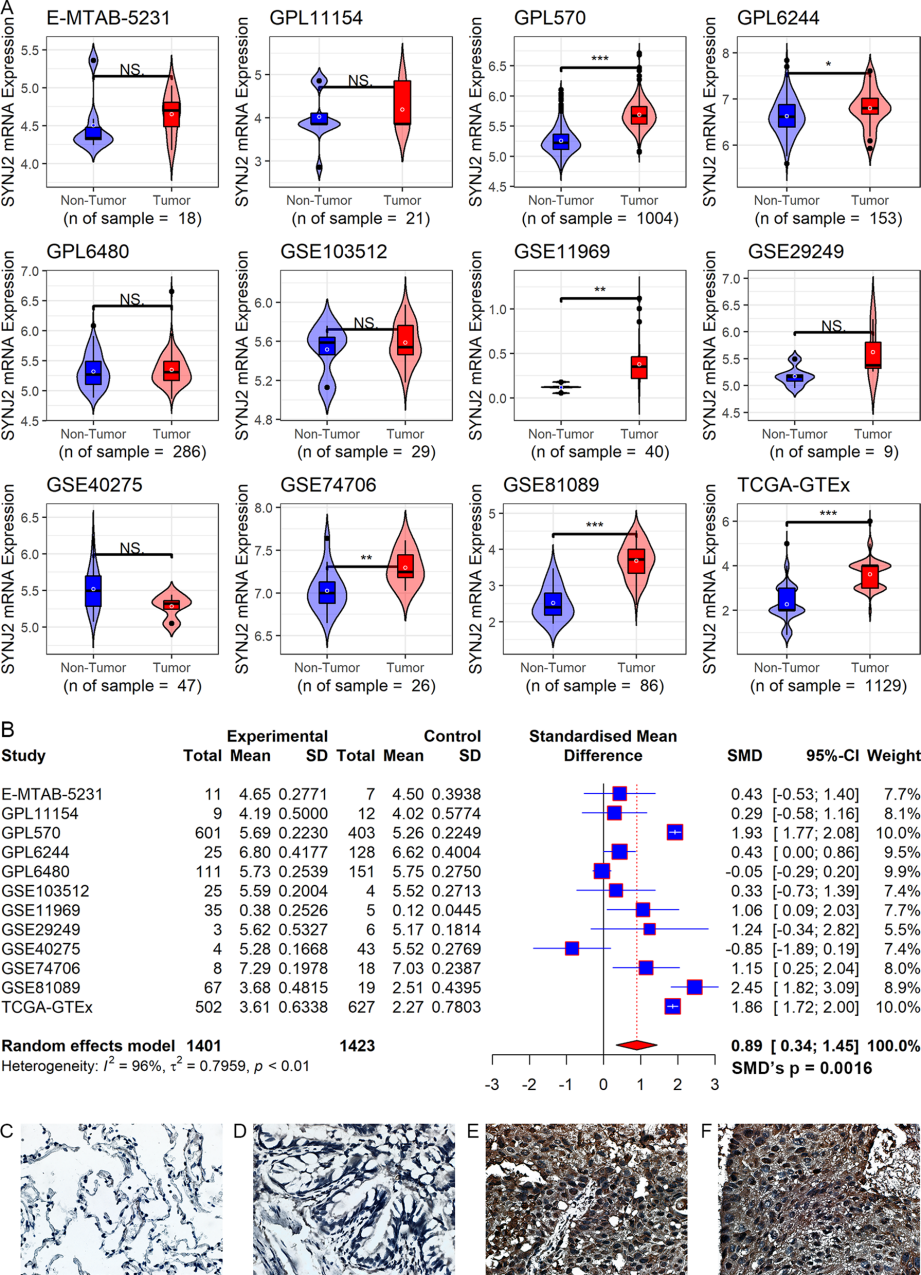
SYNJ2 expression between the LUSC group and control group. **A** The mRNA expression of *SYNJ2* between the LUSC and control groups. *p < 0.05; **p < 0.01; ***p < 0.001; *p* values are based on the Wilcoxon rank-sum test or *t*-test. **B** The forest plot of mRNA expression of *SYNJ2* between the LUSC and control groups. **C** The protein levels of SYNJ2 between the LUSC tissues (**C**, **D**) and normal lung tissues (**E**, **F**)

**Fig. 3:**
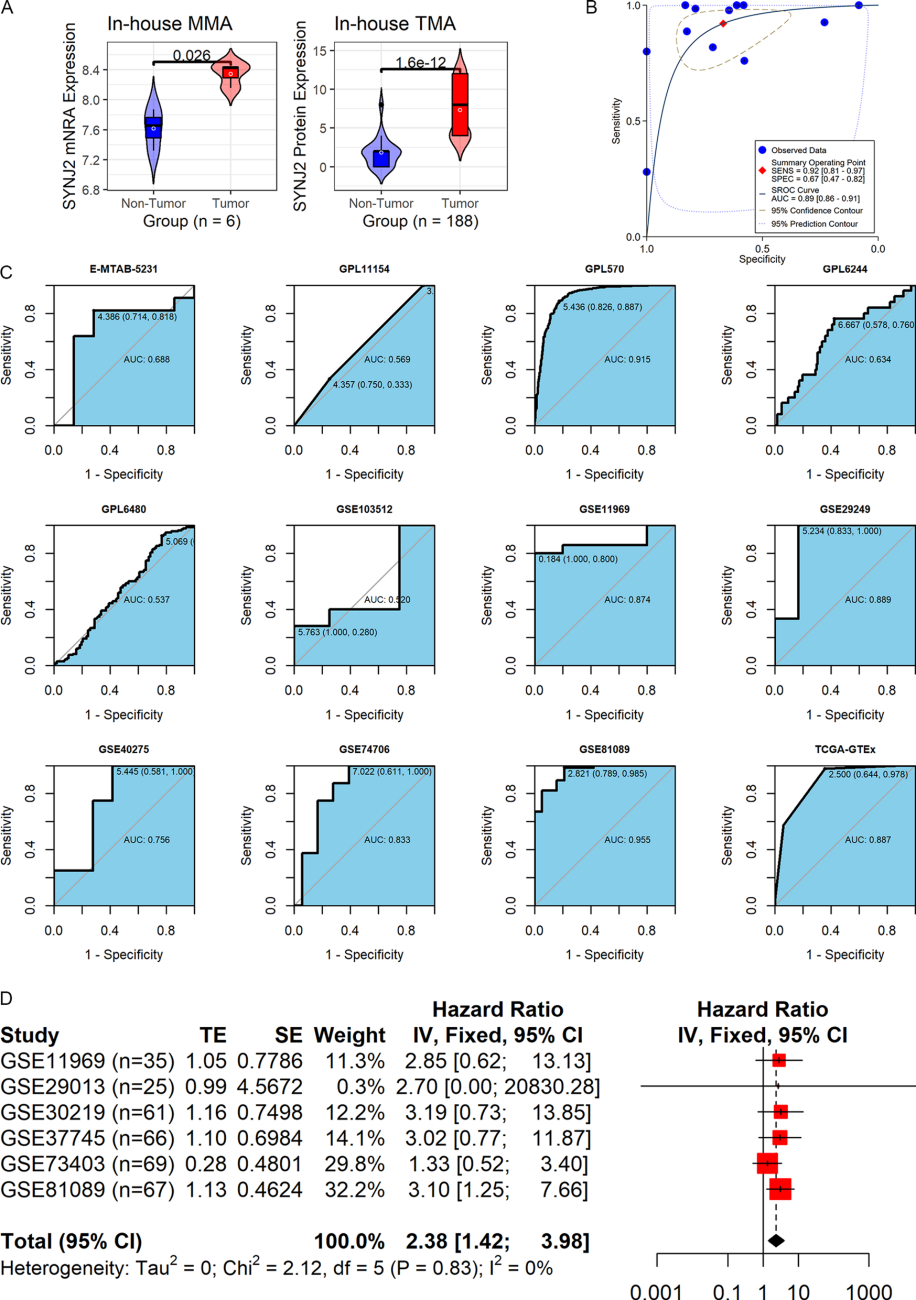
The expression, prediction ability, and prognosis relationship of SYNJ2 in LUSC. **A** The mRNA and protein levels of SYNJ2 expression in LUSC; The *p* values on the top of boxes are based on the Wilcoxon rank-sum test. **B**, **C** The prediction ability of *SYNJ2* expression for LUSC. **D** *SYNJ2* expression is related to the prognosis of patients with LUSC based on univariate Cox regression analysis results

The statistical significance of the *SYNJ2* expression difference between the LUSC and control groups was detected in 6 of 12 merged datasets included in this study, and 11 (except for “GSE40275”) of the 12 datasets indicated the *SYNJ2* mRNA expression was higher in the LUSC group than that in the control group (Fig. 2A). Moreover, an integrative analysis including 2824 samples also suggested overexpression of *SYNJ2* in LUSC (SMD = 0.89, 95% CI 0.34–1.45) (Fig. 2B), and there was no publication bias in the SMD results (*Begg’s* test, *p* = 0.784) (Additional file 4). The sensitivity analysis also determined the robustness of SMD results, as the results and heterogeneity of SMD did not change significantly after deleting any merged datasets (Additional file 5). At protein levels, it can be seen from Fig. 2C–F that SYNJ2 protein was significantly detected in not normal lung tissues (Fig. 2 C, D) but LUSC tissues (Fig. 2E, F). Further, by analyzing in-house samples, the upregulated SYNJ2 expression at both mRNA and protein levels in LUSC was validated (*p* < 0.05; Fig. 3A).

### Clinical relevance of SYNJ2 mRNA expression in LUSC

The sROC analysis revealed a high accuracy of *SYNJ2* mRNA expression in distinguishing LUSC samples from non-LUSC samples (AUC = 0.89; Fig. 3B). At least moderate accuracy of *SYNJ2* mRNA expression identifying LUSC was detected in seven datasets (AUC > 0.75; Fig. 3C).

A total of six datasets involving 323 cases were obtained for the prognostic analysis of *SYNJ2* in LUSC. The results showed that patients with increased *SYNJ2* expression tended to have poorer OS than those with decreased *SYNJ2* expression (HR = 2.38, 95% CI 1.42–3.98) (Fig. 3D).

### Biofunctions and mechanisms prediction of SYNJ2 in LUSC

**Fig. 4:**
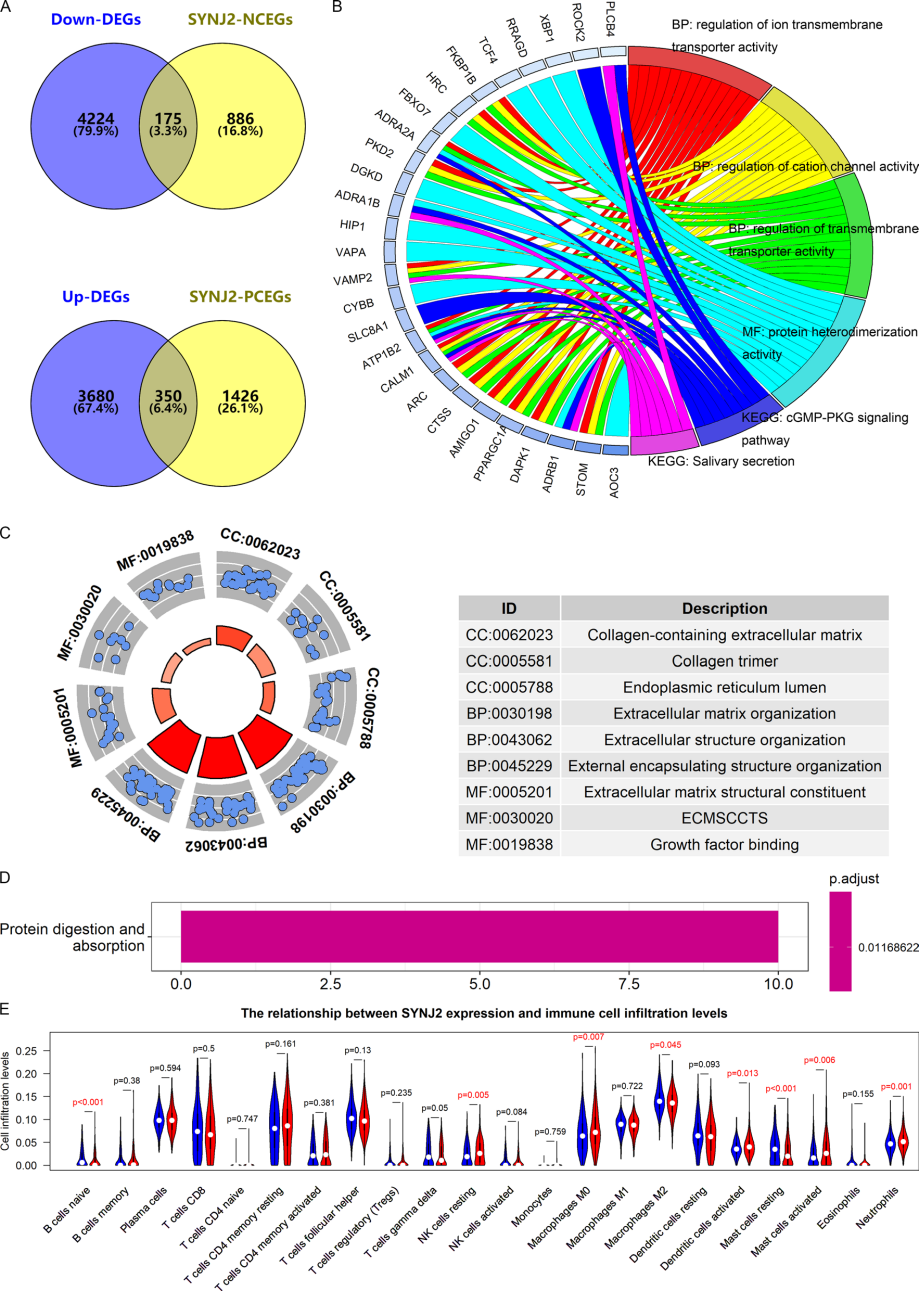
Biofunctions and mechanisms prediction of *SYNJ2* in lung squamous cell carcinoma. **A** Selection of downregulated negatively co-expressed genes (Down-NCEGs) and upregulated positively co-expressed genes (Up-PCEGs). **B** Gene ontology analysis and Kyoto Encyclopedia of Genes and Genomes (KEGG) analysis of Down-NCEGs. **C** Gene ontology analysis of Up-PCEGs. **D** KEGG analysis of Up-PCEGs. **E** The relationship between *SYNJ2* expression and infiltration levels of immune cells; the blue “violin” refers to the low-*SYNJ2* expression group, while the red “violin” refers to the high-*SYNJ2* expression group

A total of 350 Up-PCEGs and 175 Down-NCEGs were identified (Fig. 4A). The analyses of gene ontology and KEGG showed that: (1) via Down-NCEGs, *SYNJ2* may affect the regulation of ion transmembrane transporter activity (biological process), protein heterodimerization activity (molecular function), and cGMP-PKG signaling pathway (KEGG signaling pathway) (Fig. 4B). (2) based on Up-PCEGs, *SYNJ2* was involved in cell components such as collagen, biological processes such as extracellular matrix organization; molecular functions such as extracellular matrix structural constituent (Fig. 4C); and signal pathways, such as protein digestion and absorption and extracellular matrix-receptor interaction (Fig. 4D).

It can be seen from Fig. 4E that LUSC samples with *SYNJ2* higher expression were detected with more resting NK cells, M0 macrophages, activated dendritic cells, activated mast cells, and neutrophils and less naive B cells, M2 macrophages, and resting mast cells.

### Different SYNJ2 expression and its clinical relevance in various cancers

**Fig. 5:**
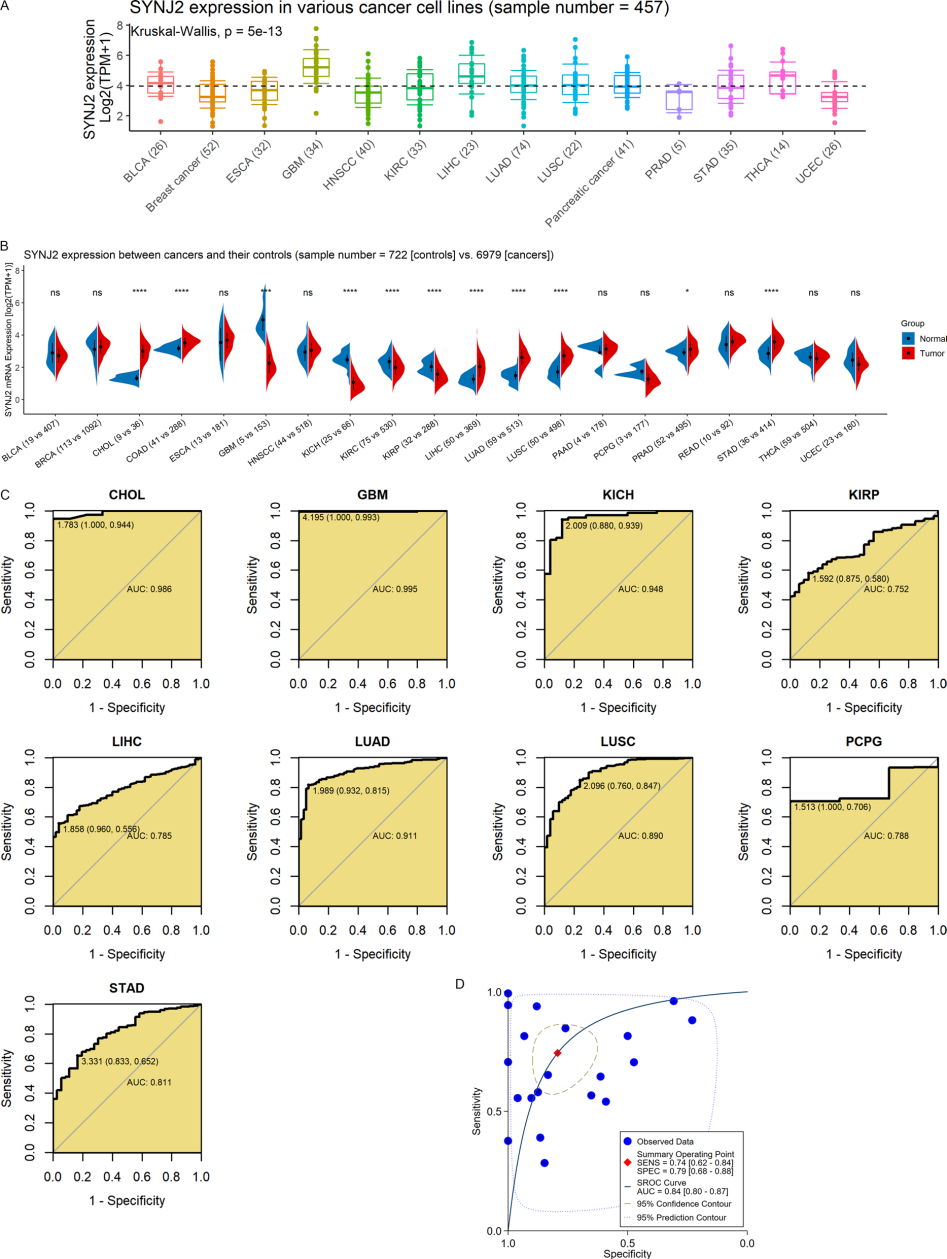
*SYNJ2* expression in pan-cancer and its prediction ability for multiple cancers. **A** *SYNJ2* expression in various cancer cell lines. **B** Differential expression of *SYNJ2* between normal cancer tissues; ^ns^*p* > 0.05; *p < 0.05; **p < 0.01; ***p < 0.001; *p*-value is based on the Wilcoxon rank-sum test. **C** The prediction ability of *SYNJ2* expression for multiple cancers. **D** The prediction ability of *SYNJ2* expression for pan-cancers

Differential expression of *SYNJ2* in LUSC was identified, while its expression in multiple cancers and clinical relevance had not been discussed before. In this study, various *SYNJ2* expression was identified between 14 cancer cell lines (Fig. 5A). By analyzing 9781 samples, compared to normal tissues, *SYNJ2* was upregulated in cholangiocarcinoma (CHOL), colon adenocarcinoma (COAD), liver hepatocellular carcinoma (LIHC), lung adenocarcinoma (LUAD), LUSC, prostate adenocarcinoma (PRAD), and stomach adenocarcinoma (STAD) and downregulated in glioblastoma multiforme (GBM), kidney chromophobe (KICH), kidney renal clear cell carcinoma (KIRC), and kidney renal papillary cell carcinoma (KIRP) (*p* < 0.05; Fig. 5B). *SYNJ2* expression made it feasible to differentiate cancer samples from corresponding normal samples with at least moderate accuracy (AUC > 0.75; Fig. 5C). The results of sROC analysis also demonstrate high accuracy of *SYNJ2* expression in identifying 20 types of cancers from their controls (AUC = 0.87; Fig. 5D).

**Fig. 6:**
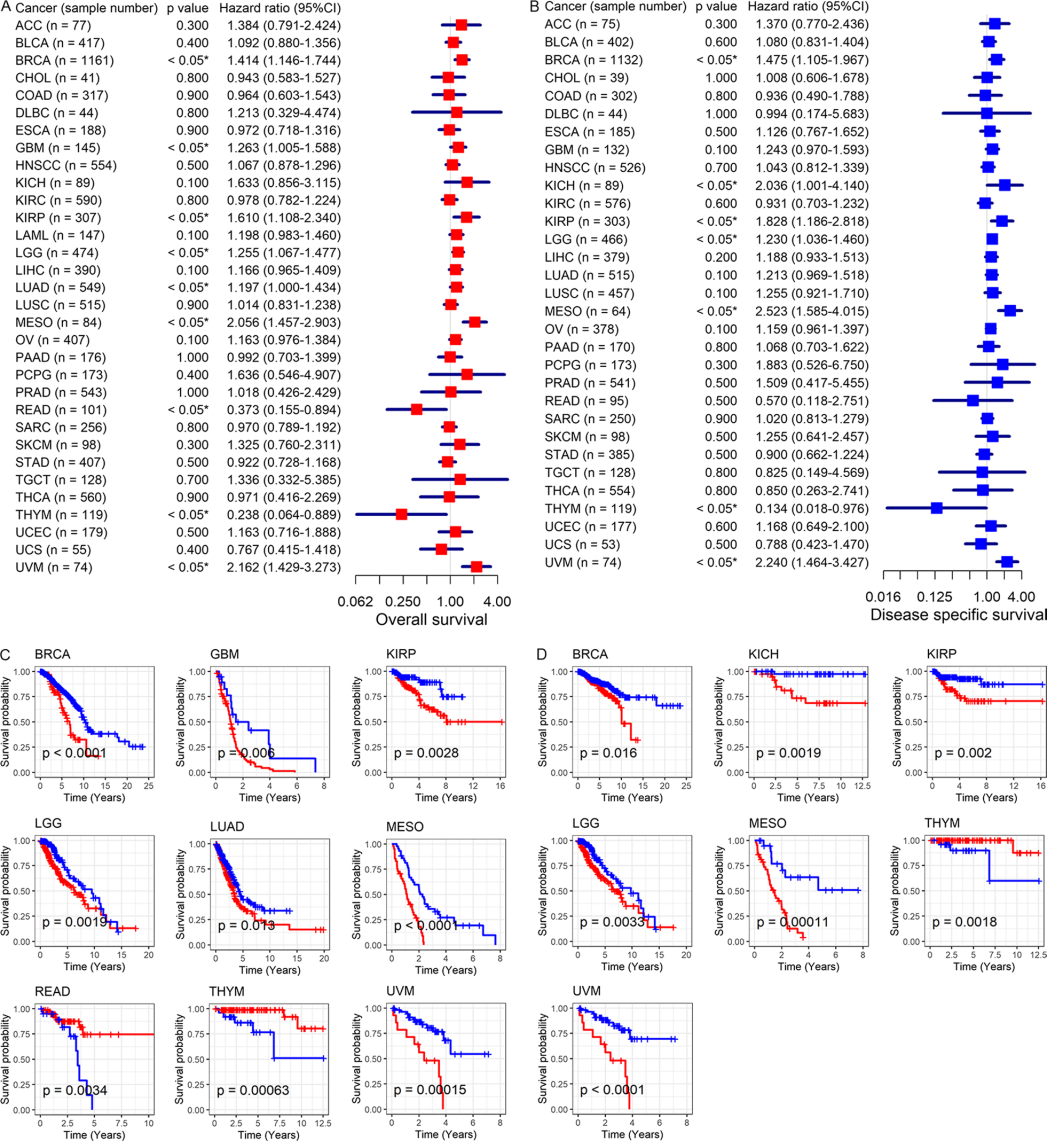
Relation of *SYNJ2* expression with overall survival and disease-specific survival of cancers patients. **A**, **B** *SYNJ2* expression is related to overall survival (**A**) and disease-specific survival (**B**) of cancers patients based on the results of univariate Cox regression analysis. **C**, **D** *SYNJ2* expression is related to overall survival (**C**) and disease-specific survival (**D**) of cancers patients based on the results of Kaplan–Meier curves

**Fig. 7:**
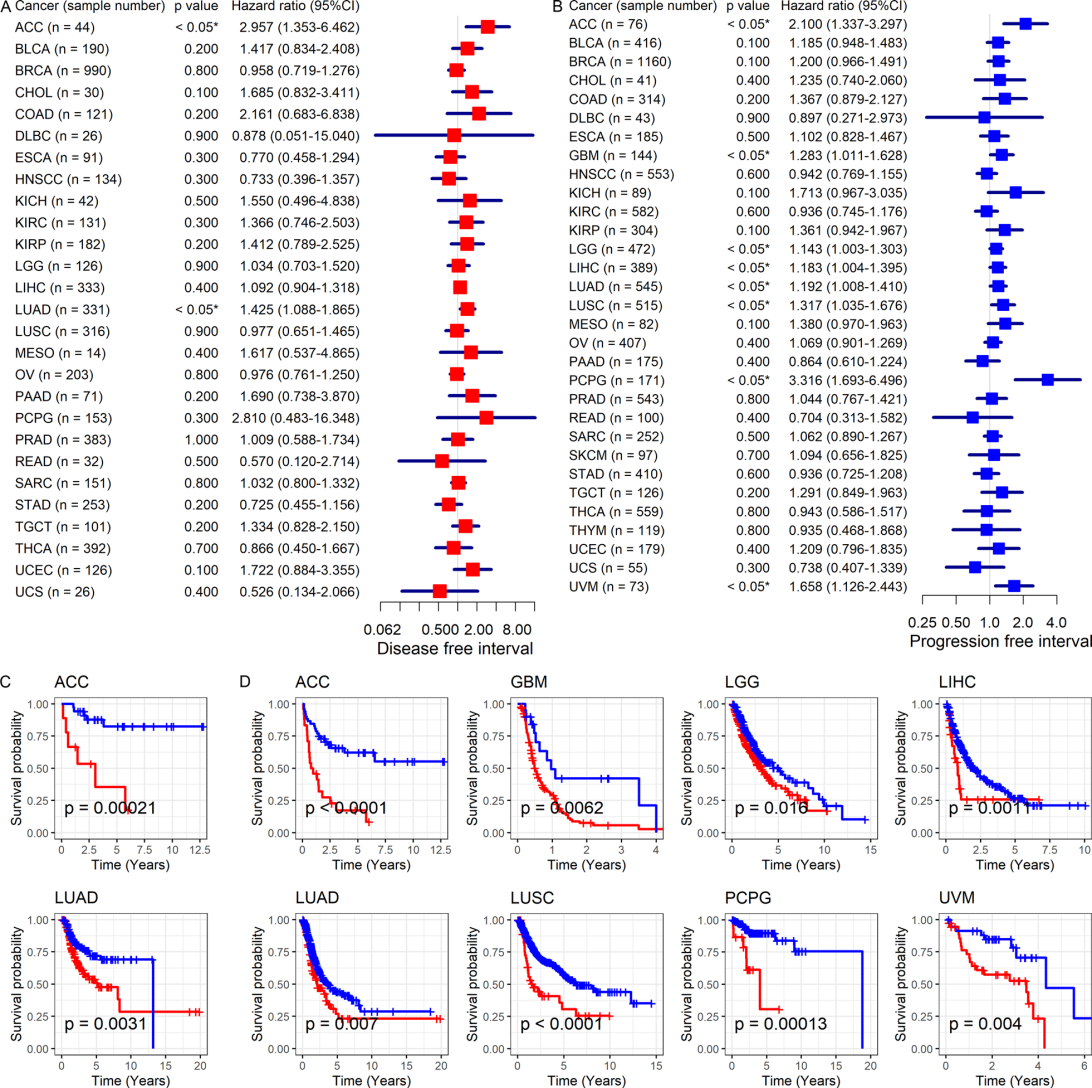
Relation of *SYNJ2* expression with disease-free interval and progression-free interval of cancers patients. **A**, **B** *SYNJ2* expression is related to disease-free interval (**A**) and progression-free interval (**B**) of cancers patients based on the results of univariate Cox regression analysis. **C**, **D** *SYNJ2* expression is related to disease-free interval (**C**) and progression-free interval (**D**) of cancers patients based on the results of Kaplan–Meier curves

According to Cox analyses and Kaplan–Meier curves, *SYNJ2* expression was associated with the prognosis of several cancers. In detail, high expression of *SYNJ2* was related to the poor OS of patients with breast invasive carcinoma (BRCA), GBM, KIRP, LGG (brain lower grade glioma), LUAD, mesothelioma (MESO), and uveal melanoma (UVM) and poor disease specific survival (DSS) of patients with BRCA, KICH, KIRP, LGG, MESO, and UVM (hazard ratio [HR] > 1, *p* < 0.05; Fig. 6A–D). On the contrary, increased *SYNJ2* expression was relevant to the good OS and DSS of THYM patients (HR < 1, *p* < 0.05; Fig. 6A–D). Moreover, upregulated expression of *SYNJ2* represented unfavorable disease free interval (DFI) and progression free interval (PFI) for patients with adrenocortical carcinoma (ACC) and LUAD (HR > 1, *p* < 0.05; Fig. 7A–D); it also served as a risk factor for PFI of patients with GBM, LGG, LIHC, LUSC, pheochromocytoma and paraganglioma (PCPG), and UVM (HR > 1, *p* < 0.05; Fig. 7B, D). Notably, even though *SYN2* expression was not found significantly correlated with the prognosis for LUSC in terms of OS, DSS, and DFI in the single TCGA dataset (Figs. 6, 7), the patients with elevated *SYNJ2* expression had an unfavorable OS based on multiple datasets and 323 samples (Fig. 3D), and thus *SYNJ2* was ultimately considered a risk factor for the prognosis of LUSC patients.

### Mechanisms prediction of SYNJ2 and the immune relevance of SYNJ2 in pan-cancer

**Fig. 8:**
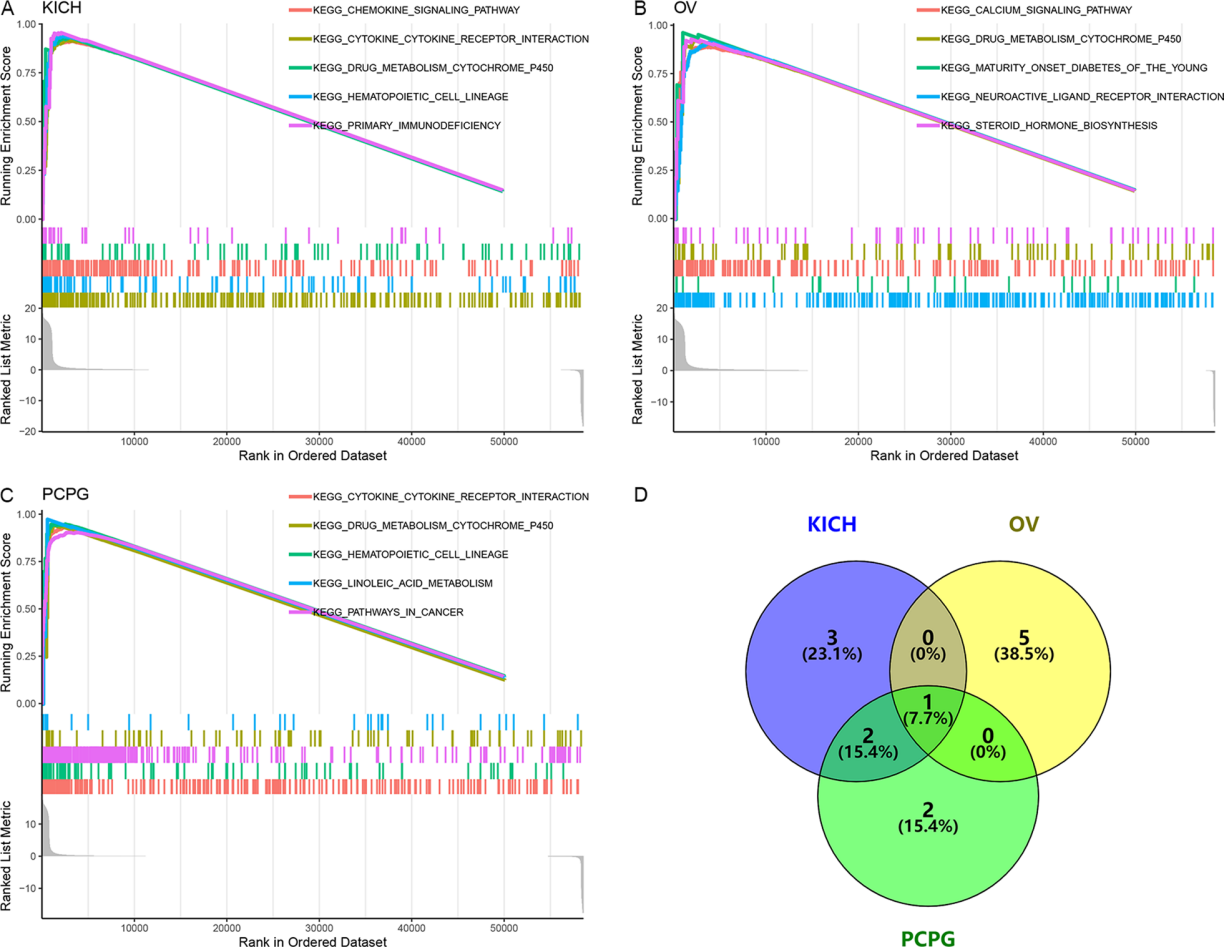
Gene set enrichment analyses of *SYNJ2* in multiple cancers

In three cancers—KICH, OV (ovarian serous cystadenocarcinoma), and PCPG, *SYNJ2* were found related to at least five KEGG signaling pathways (Fig. 8A–C). Interestingly, “DRUG METABOLISM CYTOCHROME P450” was the overlap of three cancers (Fig. 8D), indicating that the important mechanism of *SYNJ2* in various cancers may be related to this pathway.

**Fig. 9:**
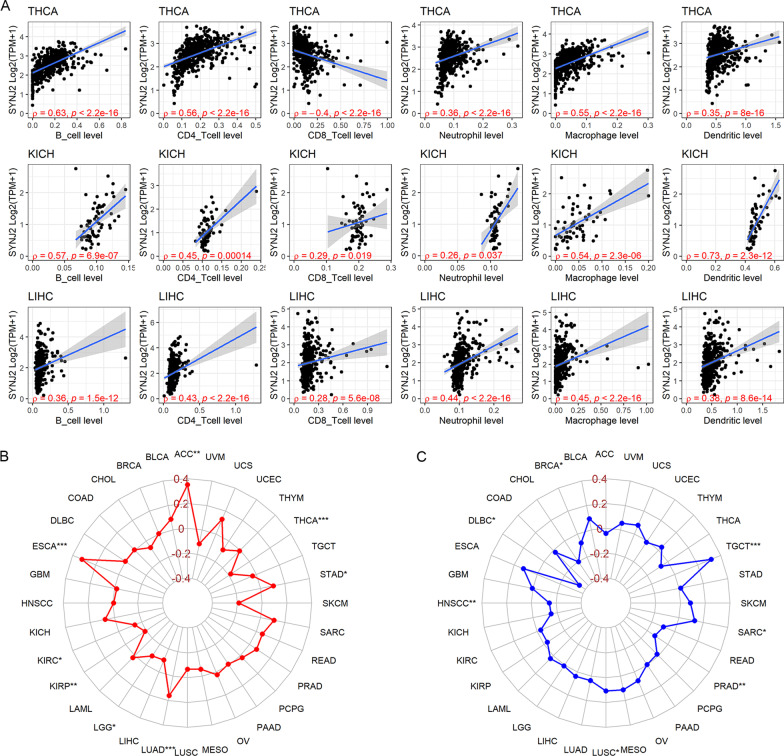
Immune relevance of *SYNJ2* in pan-cancer. **A** The relationship between *SYNJ2* expression and infiltration levels of all six immune cells. **B**, **C** Spearman coefficient of *SYNJ2* expression with tumor mutational burden (**B**) and microsatellite instability (**C**)

**Fig. 10:**
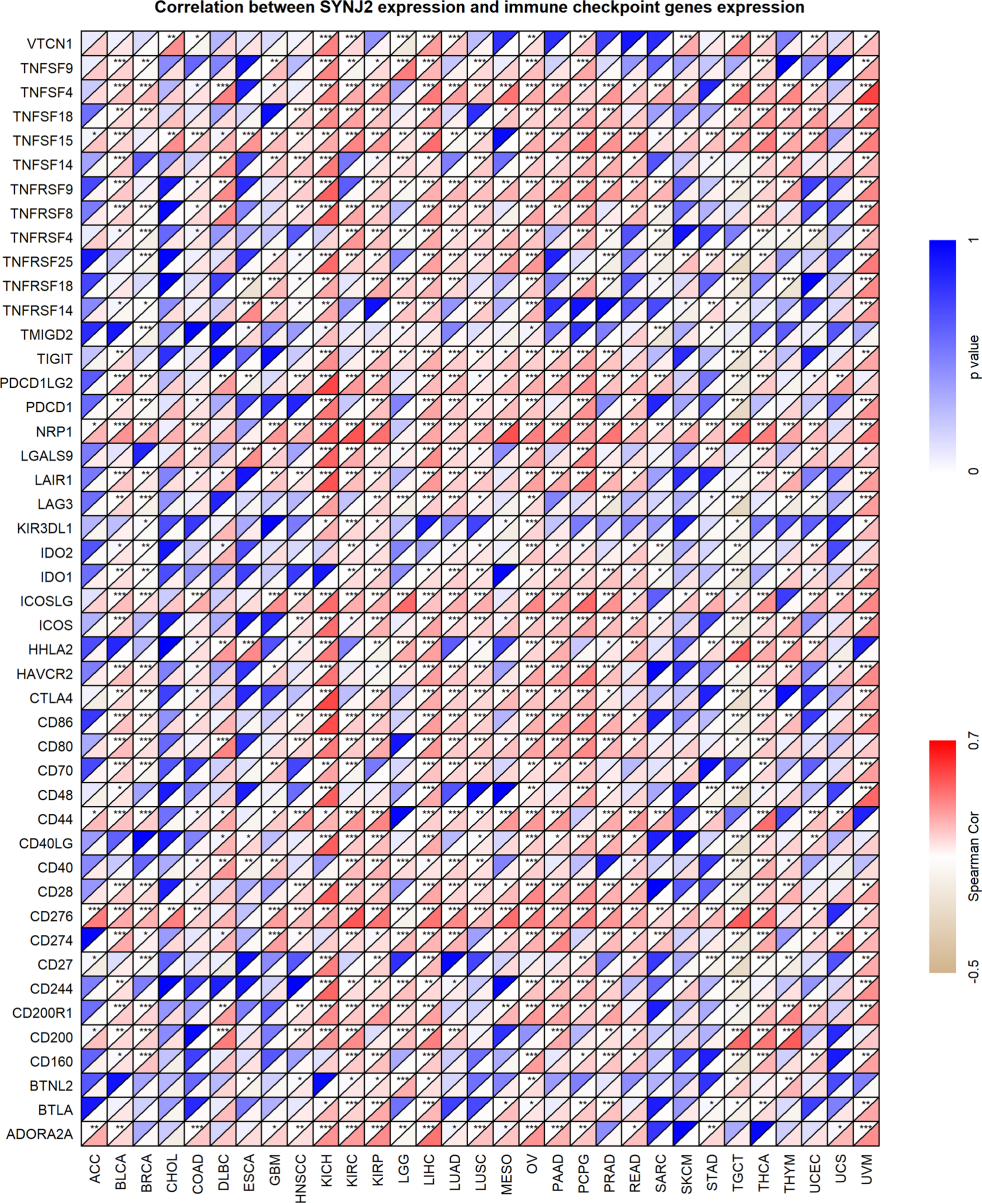
The expression relevance between *SYNJ2* and immune checkpoints

The immune response plays an essential role in anti-tumor, and thus the immune relevance of *SYNJ2* in pan-cancer was investigated in this study. It can be viewed from Fig. 9A that *SYNJ2* expression was positively (except for CD8 T cell in THCA) and closely related to infiltration levels of six immune cells in THCA, KICH, and LIHC. Moreover, the expression level of *SYNJ2* was positively associated with TMB in ACC, esophageal carcinoma (ESCA), LUAD, and STAD; it was negatively related to TMB in KIRC, KIRP, LGG, and THCA (Fig. 9B). For MSI, *SYNJ2* expression was positively relevant to LUSC, sarcoma (SARC), and testicular germ cell tumors (TGCT) and negatively associated with MSI in BRCA, DLBC (lymphoid neoplasm diffuse large B-cell lymphoma), HNSCC and prostate adenocarcinoma (PRAD) (Fig. 9C). Moreover, the significant relevance of *SYNJ2* and immune checkpoints were defined in Fig. 10. For example, *SYNJ2* demonstrated a positive association in KICH, LIHC, OV, PAAD, PCPG, and UVM and a negative association in TGCT with expression levels of not less than 15 immune checkpoints (Fig. 10).

## Discussion

For the first time in this field, this study identified the expression and roles of *SYNJ2* in LUSC. Based on the analysis of 3018 samples, SYNJ2 was significantly upregulated in LUSC at both mRNA and protein levels, and using multicenter samples, overexpressed *SYNJ2* predicted poor prognosis for LUSC patients. The cancer-promoting effect of *SYNJ2* may be related to protein digestion and absorption and extracellular matrix-receptor interaction. *SYNJ2* expression was closely related to immune cell infiltration, indicating its role in the immune response. Moreover, this study initially investigated the expression levels and clinical relevance of *SYNJ2* in pan-cancer, demonstrating the novel and potential biomarker for the prediction and treatment of cancers.

*SYNJ2* is a synaptic binding protein with a role in endocytosis. Its involvement in human cancer has not been well-studied [6, 31]. Previous studies have reported that *SYNJ2* is responsible for tumor initiation and progression. With genetic changes, *SYNJ2* triggers colorectal cancer [10], medulloblastoma [9], prostate cancer [11], and hepatocellular carcinoma [32]. Overexpressed *SYNJ2* boosts the invasion and migration of glioma [13] and breast cancer cells and prompts poor prognosis for cancer patients [14].

However, only one study by Ben-Chetrit et al. [14] has focused on the relationship between *SYNJ2* and lung cancer. To verify the correlation between *SYNJ2* and tumors, Ben-Chetrit et al. used an online informatics tool to show that high *SYNJ2* transcript levels indicated a shorter lifespan for patients with lung cancer. Yet the study used only single-source data and limited samples, ignored genetic background, and simply evaluated the survival time. Moreover, distinct histological subtypes of lung cancer are characterized by specific morphological and molecular phenotypes. Thus *SYNJ2* may play diverse roles in LUSC, lung adenocarcinoma, and small cell lung cancer, while the study of Ben-Chetrit et al. concentrated on all lung cancer rather than specific subtypes was not sufficient to understand the full spectrum of functions and mechanisms of action of *SYNJ2* in LUSC. Therefore, the roles of *SYNJ2* in LUSC remain largely unknown.

In this study, integrated analysis based on data covering more than 2824 samples revealed upregulated *SYNJ2* mRNA expression in LUSC. Furthermore, in-house experiments verified a higher SYNJ2 expression in LUSC tissues than in non-tumorous lung tissues at mRNA and protein levels. In addition, this research not only demonstrated that *SYNJ2* made it feasible to distinguish between LUSC and non-LUSC specimens but indicated that patients with high levels of *SYNJ2* tend to live shorter lives. Thus, *SYNJ2* was dramatically upregulated in LUSC and can predict both the cancer status and the poor prognosis for patients with LUSC, which has not been reported before, indicating the novelty of this study.

The expression patterns of *SYNJ2* in different cancers were various, and its clinical relevance is conspicuous. Distinct *SYNJ2* expression levels were identified in 14 types of cancer cell lines. Previous research showed differential expression levels of *SYNJ2* between some cancers (e.g., BRCA [14] and LIHC [32]) and their control samples, and our study further comprehensively analyzed 20 cancers and revealed that increased and decreased *SYNJ2* expression levels were defined in seven cancers (CHOL, COAD, LIHC, LUAD, LUSC, PRAD, and STAD) and four cancers (GBM, KICH, KIRC, and KIRP), respectively. *SYNJ2* expression made it feasible to differentiate multiple types of cancers from their corresponding normal samples with at least moderate accuracy, suggesting its potential in identifying cancer status. In addition, elevated *SYNJ2* expression was associated with the unfavorable prognosis (at least one of OS, DSS, DFI, and PFI) of cancers with ACC, BRCA, GBM, KICH, KIRP, LGG, LIHC, LUAD, LUSC, MESO, PCPG, and UVM, while it represented favorable OS and DSS of THYM patients. From these results, *SYNJ2* generally served as a risk marker for the prognosis of cancer patients. Taken together, *SYNJ2* may be a novel marker for predicting cancer status and prognosis of multiple cancers.

Few studies have focused on the molecular mechanistic understanding of *SYNJ2* in human malignant tumors. According to previous studies, high expression of *SYNJ2* was correlated with hepatocellular tumorigenesis via the CTCF/POLR2A-*SYNJ2* axis [32] and with metastasis of glioma [13] and breast cancer through regulating the formation of lamellipodia and invadopodia [14]. In addition to these, *SYNJ2* negatively regulated the PI3K/Akt pathway and was necessary for vesicle transport, focal adhesion, and lamellipodia as well as invadopodia formation, which led to cell survival, proliferation, invasion, and migration [14, 31]. Indeed, a similar finding can also be found in our study on LUSC (the proteins encoded by Up-PCEGs of *SYNJ2* gathered in the extracellular matrix-receptor interaction). Our study also revealed that *SYNJ2* might affect protein digestion and absorption (*SYNJ2* protein is known to interact with the corresponding substrate, resulting in the translocation of the encoded protein to the plasma membrane and thereby inhibiting the clathrin-mediated endocytosis [32]). These findings implied that the roles *SYNJ2* played in LUSC may be related to extracellular matrix-receptor interaction and protein metabolism; however, it requires further experiment exploration and verification. In addition, *SYNJ2* was found related to the “DRUG METABOLISM CYTOCHROME P450” KEGG signaling pathway in several cancers (at least KICH, OV, and PCPG), indicating its druggable potential, which has also been demonstrated in BRCA before [14].

Little research has focused on the relevance between *SYNJ2* and immunity before. However, based on the results of our study, *SYNJ2* likely participated in the immune response by affecting filtration levels of immune cells, the mechanisms of which were complex with the fact that: (1) The infiltration levels of several antigen presenting cells (e.g., activated dendritic cells and neutrophils [33–37]), were significantly increased in LUSC samples with higher expression levels of *SYNJ2*. (2) *SYNJ2* was positively associated with both infiltration levels of innate and acquired immune cells and expression levels of immune checkpoints in some cancers such as KICH and LIHC. (3) TMB and MSI were known to contribute to the production of new immune antigens and induction and promotion of immune responses [38, 39], and *SYNJ2* showed its relationship with TMB and MSI in some cancers, implying its trigger role in this field. (4) Drugs targeting immune checkpoint block are promising in the treatment of cancers [40, 41]; *SYNJ2* expression was significantly positively related to plenty of immune checkpoints in several cancers, suggesting that it may have similar clinical potential as immune checkpoints. Taken together, *SYNJ2* was closely associated with the immune microenvironment and may serve as a potential marker of immune target treatment.

There are some limitations to this study. First, the number of in-house cohorts was small and must be expanded to include more clinical samples. Second, most of the in-house cases included in our study were at an early stage, did not have distant and lymph node metastases, and lacked clinical follow-up information, which limited our capacity to gain a comprehensive understanding of the clinical significance of *SYNJ2* in LUSC. Last, a series of in vivo and in vitro experiments are needed to further explore the molecular mechanisms of action of *SYNJ2* in LUSC. More scientific experiments in the future are required to address these limitations.

In conclusion, this study validated that *SYNJ2* was significantly upregulated in LUSC tissues. Overexpressed *SYNJ2* identities cancer status and predicts a poor prognosis for individuals with one type of multiple cancers, including LUSC. This research also disclosed the close association between *SYNJ2* expression and the immune environment and the potential of this gene as a novel and potential biomarker for the prediction and treatment of multiple cancers, including LUSC.

## Supplementary Information

**Additional file 1**. The collection workflow of datasets for calculating SYNJ2 expression.

**Additional file 2**. Datasets collected for LUSC.

**Additional file 3**. The details of the 32 cancers in this study.

**Additional file 4**. Funel plot with Begg’s test for publication bias in the SMD.

**Additional file 5**. The sensitivity analysis determined the robustness of SMD results.

## Data Availability

The datasets supporting the conclusions of this article are available in the Gene Expression Omnibus [https://www.ncbi.nlm.nih.gov/gds/], the ArrayExpress [https://www.ebi.ac.uk/arrayexpress/], TCGA Research Network [www.cancer.gov/tcga], Genotype Tissue Expression [https://commonfund.nih.gov/GTEx], and DepMap [https://depmap.org/portal/]. Direct persistent links for each public dataset were shown in the Sheet 1 of the Additional file 2. In-house data can be obtained from the corresponding author for reasonable reasons.

## Abbreviations

LUSC: Lung squamous cell carcinoma
SYNJ2: Synaptojanin 2
TCGA: The Cancer Genome Atlas
IHC: Immunohistochemistry
ROC: Receiver operator characteristic
HR: Hazard ratio
KEGG: Kyoto Encyclopedia of Genes and Genomes
OS: Overall survival
DEG: Differential expression gene
SMD: Standardized mean difference
AUC: Area under the curve
Up-DEGs: Upregulated differential expression genes
Down-DEG: Downregulated differential expression gene
SYNJ2-PCEG: Positively co-expressed gene of SYNJ2
SYNJ2-NCEG: Negatively co-expressed gene of SYNJ2
Up-PCEG: Upregulated positively co-expressed gene
Down-NCEG: Negatively co-expressed gene.
TMB: Tumor mutational burden
MSI: Microsatellite
CI: Confidence interval
DSS: Disease specific survival
DFI: Disease free interval
PFI: Progression free interval
