# Supplementary figures and images for: SYNJ2 is a novel and potential biomarker for the prediction and treatment of cancers: from lung squamous cell carcinoma to pan-cancer
Source: BMC Med Genomics. 2022 May 17;15:114. doi: 10.1186/s12920-022-01266-0 (PMC9112447; doi:10.1186/s12920-022-01266-0)

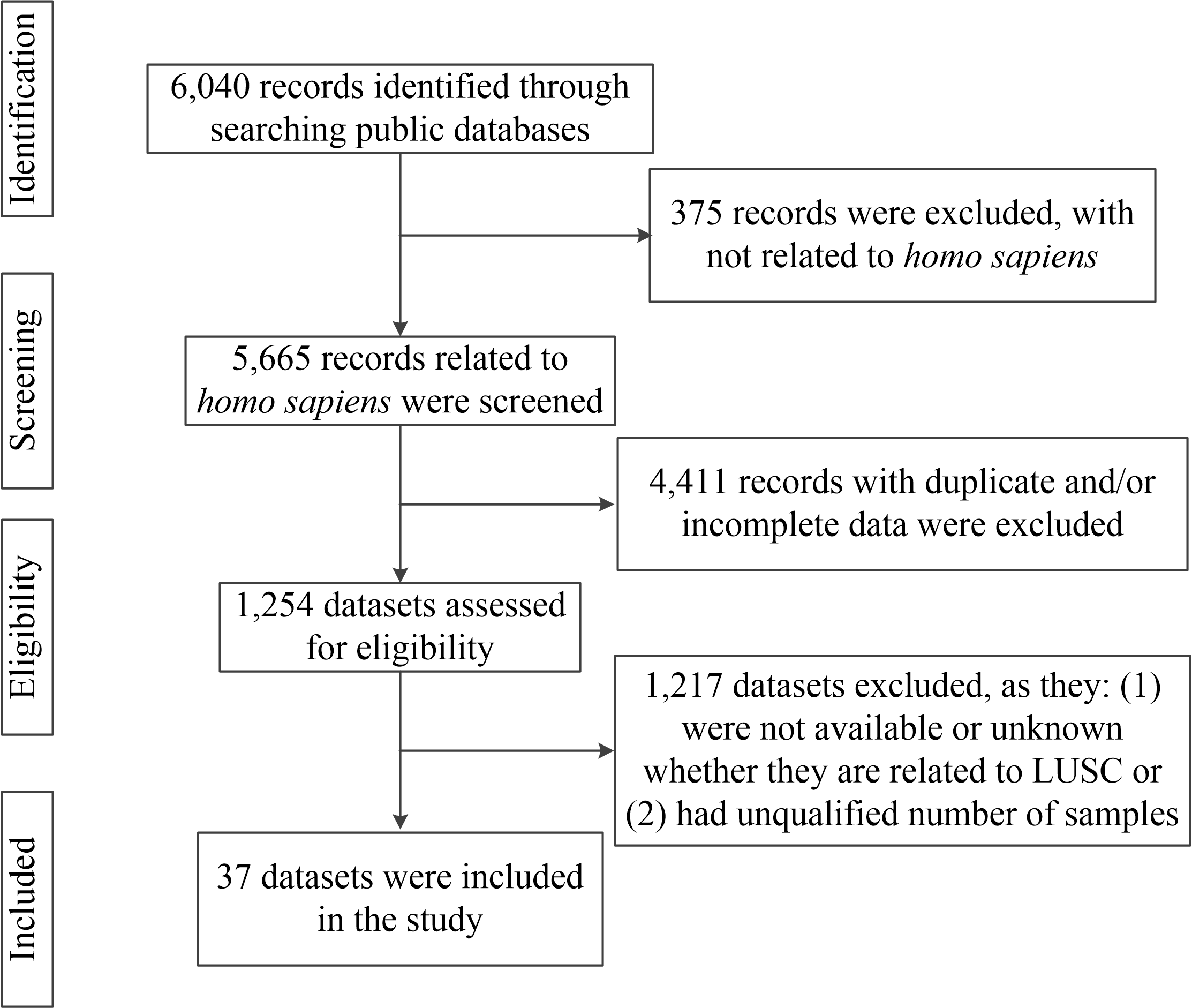

Supplement: Supplementary file 1 — Additional file 1. The collection workflow of datasets for calculating SYNJ2 expression. [file 12920_2022_1266_MOESM1_ESM.tif]

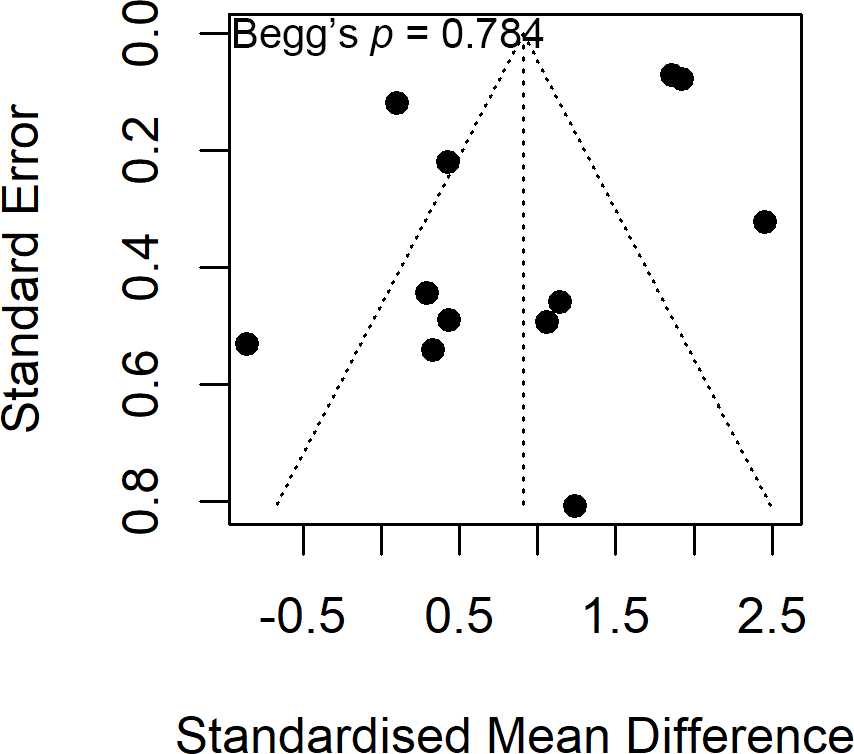

Supplement: Supplementary file 4 — Additional file 4. Funel plot with Begg’s test for publication bias in the SMD. [file 12920_2022_1266_MOESM4_ESM.tiff]
