# Supplementary material for: SYNJ2 is a novel and potential biomarker for the prediction and treatment of cancers: from lung squamous cell carcinoma to pan-cancer
Source: BMC Med Genomics. 2022 May 17;15:114. doi: 10.1186/s12920-022-01266-0 (PMC9112447; doi:10.1186/s12920-022-01266-0)
Supplement: Supplementary file 5 — Additional file 5. The sensitivity analysis determined the robustness of SMD results. [file 12920_2022_1266_MOESM5_ESM.docx]

**Additional file 5.** The sensitivity analysis determined the robustness of SMD results.

| Study | SMD | 95%CI of SMD | *p*-value | tau^2^ | I^2^ |
| --- | --- | --- | --- | --- | --- |
| Omitting E-MTAB-5231 | 0.9491 | [0.3903-1.5080] | 0.0009 | 0.7374 | 96.10% |
| Omitting GPL11154 | 0.9638 | [0.4056-1.5220] | 0.0007 | 0.7319 | 96.10% |
| Omitting GPL570 | 0.7877 | [0.1128-1.4626] | 0.0222 | 1.1161 | 95.40% |
| Omitting GPL6244 | 0.9614 | [0.4016-1.5212] | 0.0008 | 0.7223 | 95.80% |
| Omitting GPL6480 | 1.0539 | [0.6305-1.4774] | <0.0001 | 0.0001 | 0.5924 |
| Omitting GSE103512 | 0.9552 | [0.3981-1.5123] | 0.0008 | 0.7355 | 96.10% |
| Omitting GSE11969 | 0.8965 | [0.3340-1.4589] | 0.0018 | 0.749 | 96.20% |
| Omitting GSE29249 | 0.8904 | [0.3362-1.4446] | 0.0016 | 0.7438 | 96.20% |
| Omitting GSE40275 | 1.0513 | [0.5085-1.5942] | 0.0001 | 0.6913 | 95.90% |
| Omitting GSE74706 | 0.8886 | [0.3245-1.4527] | 0.002 | 0.7515 | 96.20% |
| Omitting GSE81089 | 0.7583 | [0.1946-1.3220] | 0.0084 | 0.7407 | 96.10% |
| Omitting TCGA-GTEx | 0.7937 | [0.1027-1.4847] | 0.0244 | 1.1782 | 95.50% |
| Pooled estimate (Random effects model) | 0.9097 | [0.3729-1.4465] | 0.0009 | 0.7369 | 95.80% |

Notes: the table shows the results after omitting any single study.
